# Von Hippel–Lindau syndrome with a rare complication of dilated cardiomyopathy: a case report

**DOI:** 10.1186/s12872-022-02913-1

**Published:** 2022-11-18

**Authors:** Ming Yu, Beibei Du, Shuai Yao, Jianghong Ma, Ping Yang

**Affiliations:** 1 Department of Cardiology, China-Japan Union Hospital of Jilin University, Xiantai, Street NO.126, Jilin 130033 Changchun, China; 2 Jilin Provincial Engineering Laboratory for Endothelial Function and Genetic Diagnosis of Cardiovascular Disease, Jilin Provincial Cardiovascular Research Institute, 130031 Changchun, Jilin Province China; 3 Department of Neurology, China-Japan Union Hospital of Jilin University, Jilin Province 130031 Changchun, China

**Keywords:** Von Hippel**–**Lindau syndrome, Congestive heart failure, Dilated cardiomyopathy, Pheochromocytoma, Case report

## Abstract

**Background:**

Von Hippel**–**Lindau (VHL) syndrome is an autosomal dominant hereditary disease affecting multiple organs, with pheochromocytoma in 26% of cases. However, VHL syndrome with congestive heart failure and dilated cardiomyopathy as the primary clinical manifestations has been rarely reported.

**Case presentation:**

A 35-year-old male patient was admitted to the hospital with dyspnea. The patient had a history of cerebellar hemangioblastoma that had been resected, and a one-year history of hypertension. Echocardiography and cardiac magnetic resonance imaging demonstrated a dilated left ventricle, decreased systolic function, and nonischemic myocardial changes. Contrast-enhanced abdominal computed tomography showed pheochromocytoma, neoplastic lesions, and multiple cysts in the kidneys and pancreas. Genetic analysis revealed a missense mutation of the VHL gene, c.269 A > T (p.Asn90Ile), which was identified as the cause of the disease. Dilated cardiomyopathy and VHL syndrome type 2 were diagnosed. The patient was administered a diuretic, α-blocker, β-blocker, and an angiotensin receptor neprilysin inhibitor (ARNI), but refused pheochromocytoma resection. At the six-month follow-up, the patient was asymptomatic with improved cardiac function.

**Conclusion:**

Cardiac involvement is an atypical manifestation in VHL syndrome. Early diagnosis with genetic screening is essential for avoiding life-threatening complications associated with VHL. The management of this rare manifestation of VHL syndrome requires further investigation.

## Background

Dilated cardiomyopathy (DCM) is heterogeneous and a common cause of heart failure. The incidence of DCM is reportedly 5 to 7 per 100,000 persons per year. Based on the etiology, DCM can be classified as either primary or secondary. Secondary DCM results from systemic diseases such as autoimmune disease, metabolic endocrine disease, amyloidosis, and others. However, the severity of cardiac dysfunction varies greatly [1].

Von Hippel**–**Lindau (VHL) syndrome is a rare autosomal dominant familial tumor-related hereditary disease. It is caused by genetic mutations in the VHL (von Hippel**–**Lindau tumor suppressor) gene, located on the short arm of chromosome 3. Up to 20% of VHL syndrome cases are caused by *de novo* mutations [2]. Phenotypically, VHL syndrome can occur without or with pheochromocytoma (types I and II, respectively). More than 26% of VHL syndrome cases are accompanied by pheochromocytoma [3, 4]. However, DCM and congestive heart failure as manifestations of VHL syndrome are not common. There are limited research data on the manifestations and outcomes of VHL syndrome-induced cardiomyopathy. To improve the ability to recognize, diagnose, and treat atypical VHL syndrome, herein we report the clinical features and genetic testing of a patient with VHL syndrome predominant manifestation was congestive heart failure.

## Case presentation

A 35-year-old male patient was admitted to our hospital with progressive chest tightness, dyspnea, and palpitations. The patient’s chest tightness and shortness of breath began 3 days before presentation and worsened upon exertion. This was accompanied by palpitations and nocturnal paroxysmal dyspnea, without chest pain, headache, or sweating. His symptoms did not improve with administration of oral theophylline. Nine years previously this patient underwent surgical resection of cerebellar hemangioblastomas, and his father had also had cerebellar hemangioblastomas removed surgically. A year before the present hospitalization, the patient acquired paroxysmal hypertension, with blood pressure up to 180/140 mmHg, but had recently achieved a blood pressure of 140/90 mmHg without medication. There was no history of diabetes or cardiovascular disease. The physical examination revealed no fever, tachycardia at 126 bpm, blood pressure 145/97 mmHg, and pulmonary rales.

**Fig. 1:**
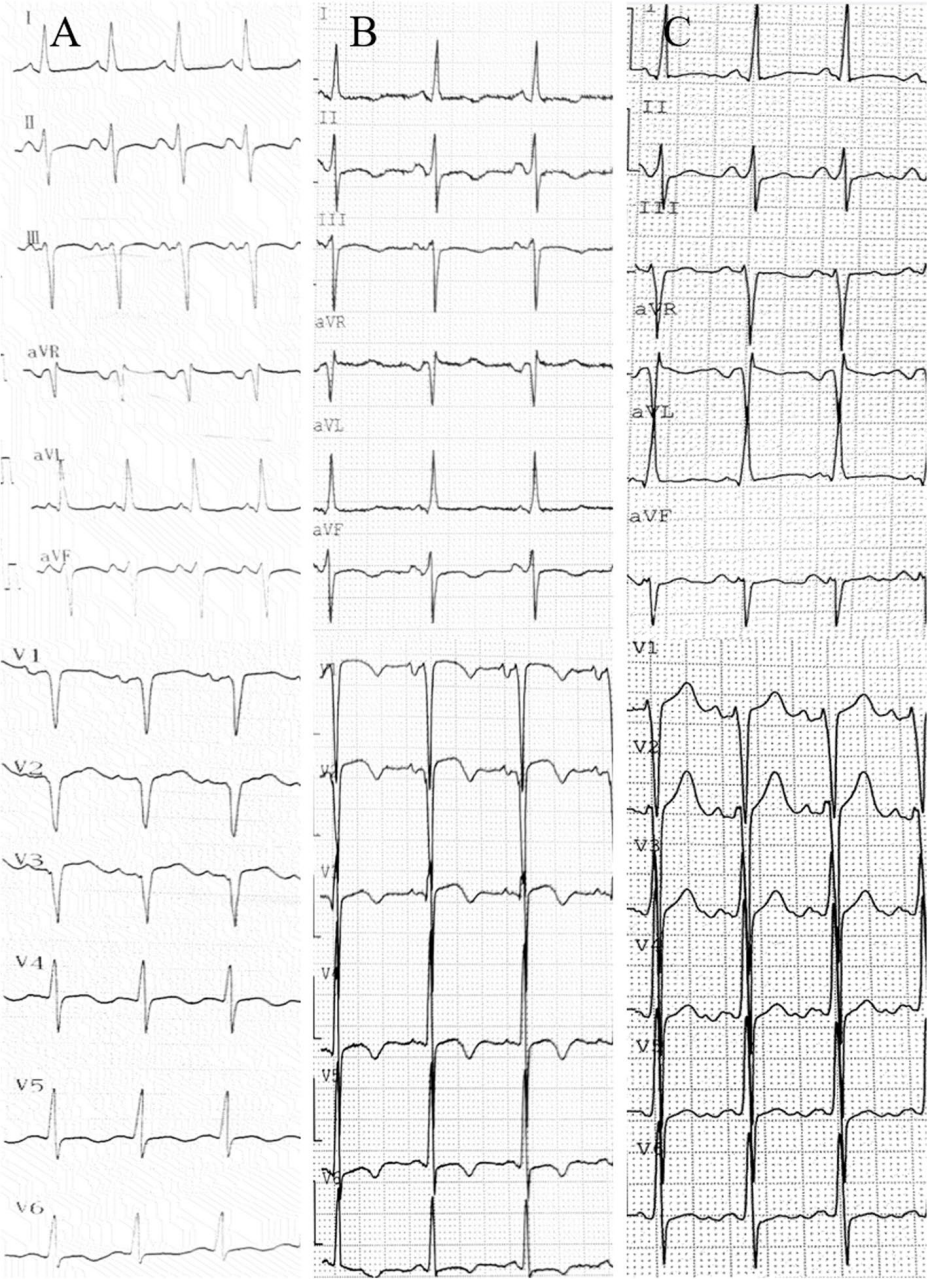
ECG examinations. **A** On admission. Sinus tachycardia with pathological Q waves and slightly elevated ST segments in leads V1-V3, and T waves inverted in leads V4-V6. **B** At discharge. T waves inverted in leads V1-V6 and I, II, III, avL, avF. **C** At 3-month follow-up. Sinus tachycardia

**Fig. 2:**
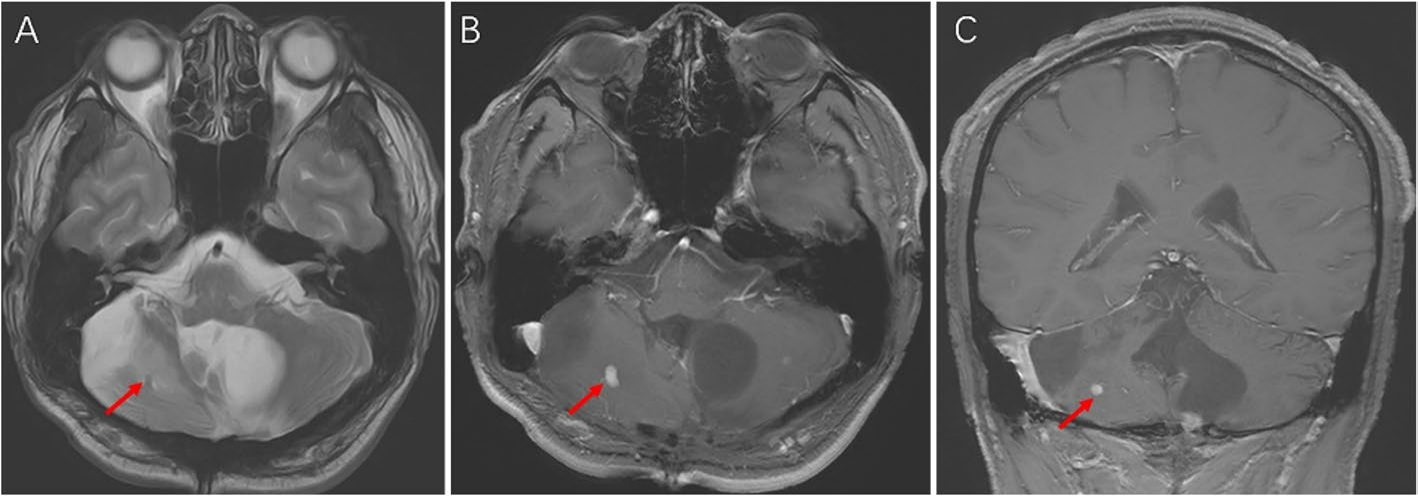
Cranial MRI. **A** T1W1. Low signal occupation in the right cerebellar hemisphere (arrow). **B** T2W1. High signal occupation in the right cerebellar hemisphere (arrow). **C** Sagittal position. Large sacs and small nodules, and mural nodules were obviously enhanced

**Fig. 3:**
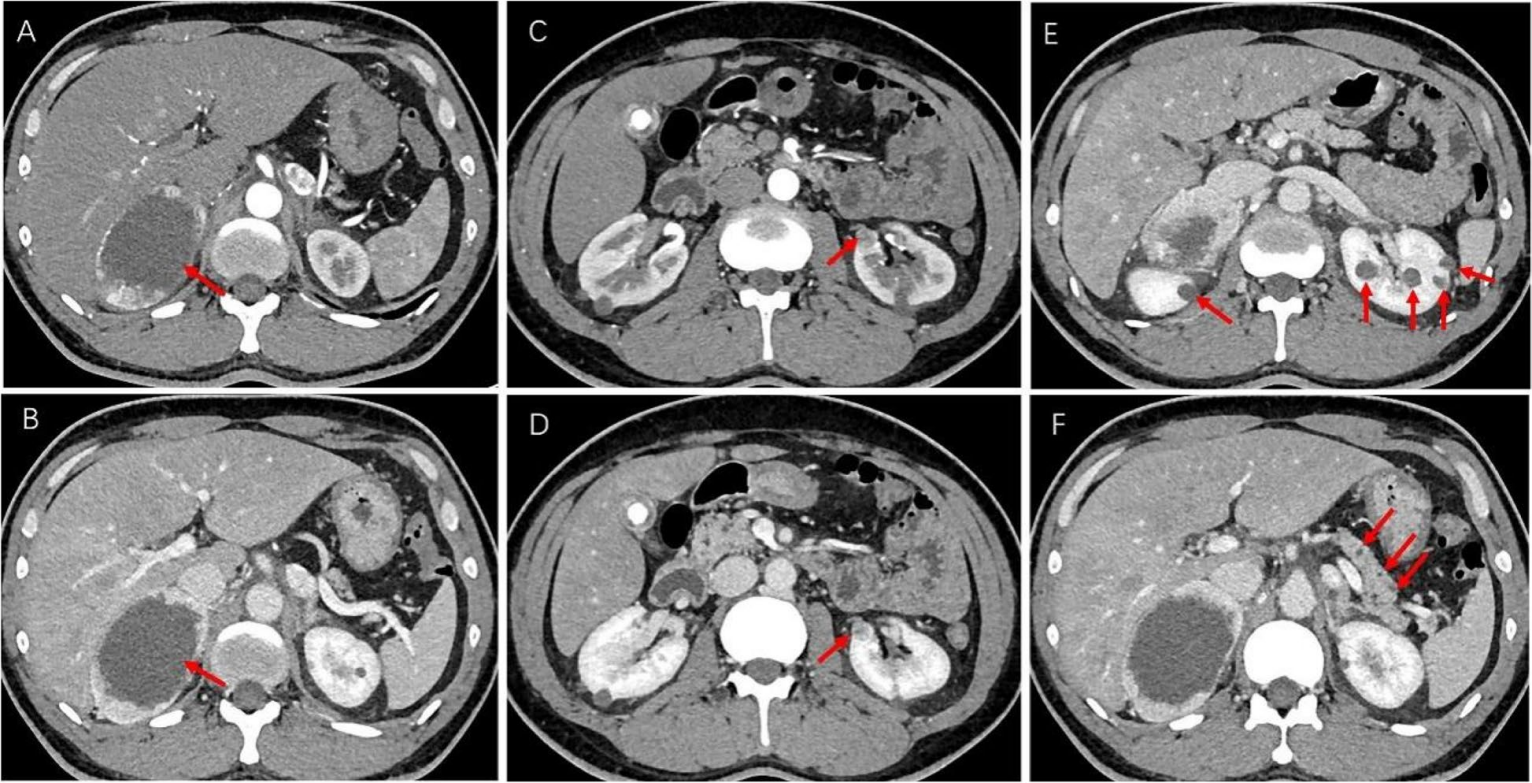
Contrast enhanced abdominal CT. **A**, **B** Pheochromocytoma (arrow) of the right adrenal gland. **A** Arterial phase. **B** Venous phase. **C**, **D** Renal carcinoma (arrow) of the left kidney. **C** Arterial phase. **D** Venous phase. **E** Multiple cysts in both kidneys (arrow). **F** Multiple cysts in pancreas (arrow)

**Fig. 4:**
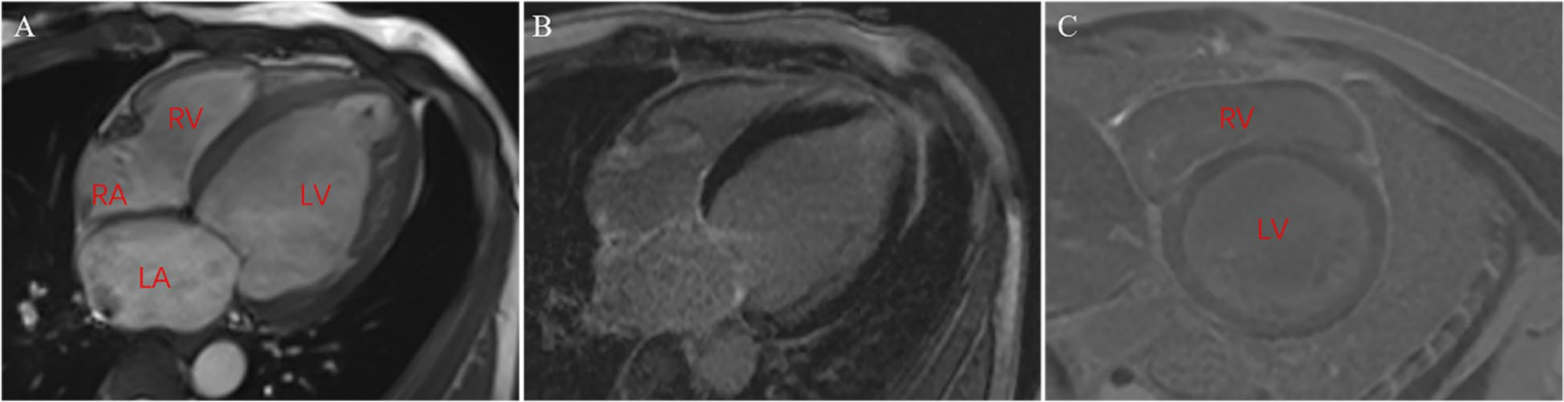
Cardiac MRI. **A** Four-chamber view showing marked LA and LV dilatation (LA: 41 mm; LV end-diastolic diameter: 78 mm; EDV: 361 ml) with highly reduced EF (EF 25%). **B**, **C** LGE image did not show significant delayed enhancement. **B** Four-chamber view. **C** Short-axis view

Upon hospital admittance, the patient’s N-terminal-pro B-type natriuretic peptide (NT-proBNP) level was significantly elevated (9920 ng/L; normal range 300–400 ng/L), but cardiac injury biomarkers and D-dimer levels were normal. Troponin I remained normal the next day. Electrocardiogram (ECG) showed sinus tachycardia with pathological Q waves, slightly elevated ST segments in leads V1-V3, and inverted T waves in leads V4-V6 (Fig. 1A). Transthoracic echocardiography showed enlargement of the left atrium (LA, 49.9 × 55.4 × 55.9 mm) and left ventricle (LV, 69 mm), diffuse LV hypokinesia, and decreased cardiac function (LV ejection fraction 23.8%). No apical ballooning changes characteristic of Takotsubo syndrome were detected. Furthermore, there was a thrombus at the apex of the LV (27.1 × 13.1 mm). Lung computed tomography (CT) indicated bilateral pulmonary edema. Cranial magnetic resonance imaging (MRI) revealed postoperative changes in the brain with multiple lesions in both cerebellar hemispheres that were consistent with the imaging findings of hemangioblastoma (Fig. 2). To identify secondary hypertension, contrast-enhanced abdominal CT was performed, which showed multiple cysts in the kidneys and the pancreas. A bilateral adrenal mass was considered most likely pheochromocytoma, and a left kidney mass, neoplastic lesions (Fig. 3). A detailed hormonal analysis was performed to assess the metabolic activity of the adrenal mass. The methoxy adrenaline level was 1.34 nmol/L (normal range < 0.5), and the methoxy norepinephrine level higher than 20 nmol/L (normal range < 0.9). The 24-hour urinary free cortisol level was slightly elevated (668.99 µg, normal range 50–437). The patient had normal aldosterone levels, whether when upright or decubitus. Further cardiac MRI showed obvious enlargement of the heart and an obvious reduction in LV systolic and diastolic function (Fig. 4A). Additionally, the trabecular muscle of the lateral wall, inferior wall, and apex of the LV was enlarged and disordered. Neither abnormal myocardial perfusion, nor abnormal enhancement with late gadolinium enhancement (LGE) was observed. These findings indicated nonischemic cardiomyopathy (Fig. 4B-C). The genetic analysis revealed a missense mutation of the VHL gene, c.269 A > T (p.Asn90Ile, Het) that resulted in the replacement of asparagine by isoleucine in the ninetieth amino acid of the gene-encoded protein.

Thus, based on the medical history, symptoms, and auxiliary examination, a diagnosis of dilated cardiomyopathy (DCM) and VHL syndrome type 2 was determined. Surgery was recommended to the patient to remove the adrenal and kidney masses, but he refused. Sacubitril valsartan (50 mg, b.i.d.), phentolamine (25 mg t.i.d.), metoprolol (47.5 mg q.d.), furosemide (20 mg b.i.d.), and spironolactone (20 mg b.i.d.) were administered. The ventricular thrombus was initially treated with subcutaneous injection of low-molecular-weight heparin, and then oral rivaroxaban for 1 month. The patient was in stable condition when discharged. ECG showed that T waves were inverted in leads V1-V6, and I, II, III, avL, and avF (Fig. 1B), and echocardiography remained unchanged. During the 3-month follow-up, the patient had no obvious chest tightness, palpitations, or dyspnea. ECG showed sinus tachycardia without ischemic changes (Fig. 1C), but echocardiography indicated no significant improvement in cardiac function. Sacubitril valsartan (100 mg b.i.d.) and metoprolol (95 mg q.d.) were prescribed at the third-month follow-up. At the 6-month follow-up, the patient had remained asymptomatic. The 6-minute walk distance was 650 m, and the NT-proBNP level was normal. Echocardiography showed that the LV end diastolic diameter was 60 mm, and LV ejection fraction was 34%. Coronary CT angiography showed no significant coronary artery stenosis.

## Discussion

This is a unique case because the patient did not present with the clinical manifestations of VHL syndrome, but rather with an unusual DCM manifestation that is rarely reported in patients with VHL syndrome.

VHL syndrome is caused by germline mutations in the VHL gene, which normally encodes a tumor suppressor protein (pVHL) involved in cellular signaling. Mutations in all three exons of the gene have been reported, leading to a variety of benign and malignant tumors of multiple organs: central nervous hemangioblastoma, retinal hemangioblastoma, pancreatic cystadenoma, pancreatic cyst, renal carcinoma, renal cyst, pheochromocytoma, and paraganglioma [5]. The incidence of VHL syndrome is estimated 1 in 36,000 live births, and the penetrance rate (i.e., the percentage of those with the mutated gene who develop VHL disease) is higher than 90% by the age of 65 years [6]. Patients with VHL syndrome have an average life expectancy of less than 49 years. The main causes of mortality have been central nervous system hemangioblastoma rupture and hemorrhage, renal cell carcinoma, and malignant hypertension caused by pheochromocytoma. For the present patient, the main manifestation was congestive heart failure, which was considered DCM, combined with cerebellar hemispheres, renal cysts, pancreatic cysts, renal carcinoma, and pheochromocytoma. VHL syndrome was highly suspected. Further genetic testing confirmed the diagnosis. For a patient with a history of cerebellar hemangioblastoma who develops heart failure, pheochromocytoma due to VHL syndrome should be considered.

Pheochromocytoma is present in 0.5% of hypertension cases, but 10 to 20% of those with both pheochromocytoma and hypertension develop heart failure [7]. DCM is an important complication of pheochromocytoma [8]. The pathogenesis of pheochromocytoma-induced DCM is widely debated. Several mechanisms have been proposed to explain myocardial dysfunction, including the direct toxic effect of catecholamine on cardiomyocytes and an imbalance of myocardial oxygen supply. Prolonged exposure to catecholamines results in interstitial fibrosis, myocardial cell apoptosis, and systolic dysfunction due to structural remodeling, leading to DCM [9]. Pheochromocytoma-induced cardiomyopathy is potentially reversible, therefore diagnosis and removal of pheochromocytoma are crucial; a delayed diagnosis may result in permanent cardiac remodeling and mortality [10]. The clinical presentations of pheochromocytoma associated with VHL syndrome differ from that of pheochromocytoma alone, and are more difficult to detect because they usually have less catecholamine secretion [4]. A PubMed search for the years 1991 through 2021 yielded 63 cases of pheochromocytoma causing DCM, of which 3 cases were comorbid with VHL syndrome [11–13]. Among the 63 patients with pheochromocytoma, 82.5% underwent pheochromocytoma resection, and of these, 96% had good ejection fraction (EF) recovery. However, some patients showed no improvement in EF after surgical removal of pheochromocytoma associated with VHL syndrome [11].

More than 500 pathogenic germline mutations have been identified in the VHL syndrome family, and type 2 VHL syndrome is characterized by missense mutations [3]. Our patient has a missense mutation of the VHL gene, c.269 A > T (p.Asn90Ile, Het), which results in the replacement of asparagine by isoleucine in the ninetieth amino acid of the gene-encoded protein. The predicted results of a missense mutation at this site on protein function are all harmful, so this mutation was considered pathogenic. The c.269 A > T (p.Asn90Ile, Het) variant identified in this patient is not novel, as three other cases of the same mutation at the same site have been reported from Denmark and China, although without descriptions of the clinical symptoms [14–16]. What is intriguing in our case is the presence of congestive heart failure, which is rarely reported in similar clinical cases of VHL syndrome. The patient’s father was given a diagnosis of cerebellar hemangioblastoma at the age of 40 years. He had no clinical manifestations of heart failure and no detectable evidence of pheochromocytoma. He refused to undergo genetic testing. It is not clear whether VHL syndrome is familial, and genetic testing by relatives is necessary to verify relevant sites, to understand the risk of the corresponding tumor.

In addition to treating any identifiable and reversible underlying cause, the management of DCM should adhere to the standard guidelines for heart failure. Management of VHL syndrome consists mainly of active surveillance, and surgical intervention when necessary. Clinicians need to weigh the risk of tumor progression and metastasis against that of surgical intervention and complications. Laparoscopic partial adrenal resection with cortex function preservation is mostly used for pheochromocytoma associated with VHL syndrome. After resection of the pheochromocytoma, cardiac dysfunction was largely eliminated [17]. The rate of resolution of cardiomyopathy generally depends on the duration of the disease. Our patient refused surgical resection of pheochromocytoma, so we administered an α-blocker, β-blocker, and ARNI to inhibit the renin-angiotensin-aldosterone system and improve the patient’s symptoms. Efficacy still requires long-term follow-up observation.

## Conclusion

Dilated cardiomyopathy in VHL syndrome is uncommon. Yet, when patients have a history of cerebellar hemangioblastomas and develop congestive heart failure, especially in the presence of intermittent hypertension, an assessment for pheochromocytoma is essential. Surgical resection of pheochromocytoma is the recommended approach for these patients, and neurohormonal blockade may be an option when surgery is not available.

## Data Availability

All relevant data supporting the conclusions of this article are included within the article.

## Abbreviations

ARNI: Angiotensin receptor neprilysin inhibitor
CT: Computed tomography
DCM: Dilated cardiomyopathy
ECG: Electrocardiography
EDV: End-diastolic volume
MRI: Magnetic resonance imaging
LA: Left atrium
LV: Left ventricle
LGE: Late gadolinium enhancement
RA: Right atrium
RV: Right ventricle
VHL: Von Hippel**–**Lindau
